# Full structural ensembles of intrinsically disordered proteins from unbiased molecular dynamics simulations

**DOI:** 10.1038/s42003-021-01759-1

**Published:** 2021-02-23

**Authors:** Utsab R. Shrestha, Jeremy C. Smith, Loukas Petridis

**Affiliations:** 1 Oak Ridge National Laboratory, Biosciences Division, UT/ORNL Center for Molecular Biophysics, Oak Ridge, TN USA; 2 Department of Biochemistry and Cellular and Molecular Biology, University of Tennessee, Knoxville, TN USA

**Keywords:** Computational biophysics, SAXS, Intrinsically disordered proteins

## Abstract

Molecular dynamics (MD) simulation is widely used to complement ensemble-averaged experiments of intrinsically disordered proteins (IDPs). However, MD often suffers from limitations of inaccuracy. Here, we show that enhancing the sampling using Hamiltonian replica-exchange MD (HREMD) led to unbiased and accurate ensembles, reproducing small-angle scattering and NMR chemical shift experiments, for three IDPs of varying sequence properties using two recently optimized force fields, indicating the general applicability of HREMD for IDPs. We further demonstrate that, unlike HREMD, standard MD can reproduce experimental NMR chemical shifts, but not small-angle scattering data, suggesting chemical shifts are insufficient for testing the validity of IDP ensembles. Surprisingly, we reveal that despite differences in their sequence, the inter-chain statistics of all three IDPs are similar for short contour lengths (< 10 residues). The results suggest that the major hurdle of generating an accurate unbiased ensemble for IDPs has now been largely overcome.

## Abstract (web summary)

Shrestha et al. show that enhancing the sampling using Hamiltonian replica exchange molecular simulation (HREMD) leads to accurate unbiased ensembles of intrinsically disordered proteins. They find that standard molecular simulation cannot reproduce small-angle scattering data as well as HREMD, highlighting the utility of the suggested approach.

## Introduction

Intrinsically disordered proteins (IDPs) exhibit biological function without folding spontaneously into a unique three-dimensional (3D) structure^1^. IDPs are abundantly present in all proteomes and play major roles in signaling, transcriptional regulation, and regulation of phase transitions in the cell via processes that may involve high-affinity interactions and recognition of partners by folding upon binding^1–6^. About 50–70% of the proteins in the human genome associated with cancers, diabetes, cardiovascular, and neurodegenerative diseases have a minimum of 30 residues that are intrinsically disordered, making IDPs possible drug targets^1^. In addition, IDPs are an essential part of plant immune signaling components and also mediate plant–microbe interactions^7^.

Understanding the function of a protein requires a determination of its 3D structure^8^. IDPs adopt highly dynamic structural ensembles, which are commonly characterized by nuclear magnetic resonance (NMR)^9^, small-angle X-ray/neutron scattering (SAXS/SANS)^10,11^, single-molecule fluorescence resonance energy transfer^12^, hydrogen-exchange mass spectrometry^13^, and circular dichroism^14,15^. However, the information content of the applied experimental techniques is insufficient to obtain the ensemble of 3D conformations an IDP adopts^16^. The experimental observables often represent averages over the ensemble and the data are typically sparse, providing too little information to unambiguously determine the 3D ensemble.

Molecular dynamics (MD) simulation can in principle provide the missing information and furnish a complete atomic resolution description of IDP structure and dynamics^2^. Recent optimizations of the protein and water potential energy functions^2,17–28^ have led to accurate simulation of short disordered peptides and model systems^18,19,29–32^. However, the simulations are not always consistent with the experiment, either because of inadequate sampling or shortcomings of the force fields^2,19,22,24,30,33,34^.

A common and successful approach to determine an IDP configurational ensemble is to force the MD results to match existing experiments, either by biasing the MD potential^35,36^ or by a posteriori reweighting the ensemble of the MD population^37,38^. One challenge for these methods is degeneracy, that is, distinct 3D conformations may yield the same observable, which may lead to overfitting. Bayesian maximum entropy optimization approaches, which aim to perturb the MD ensemble as little as possible, have been employed to avoid overfitting^35,38,39^. However, these approaches always require a prior experimental measurement and do not afford a predictive understanding of IDPs.

Recently, by enhancing the configurational sampling of MD simulations using Hamiltonian replica-exchange MD (HREMD) the configurational ensemble of an IDP was generated that is in quantitative agreement to SAXS, SANS, and NMR measurements without biasing or reweighting the simulations^40,41^. HREMD improves sampling by scaling the intraprotein and protein-water potentials^17,20^ of higher-order replicas, while keeping the potential of the lowest rank replica unscaled^42–45^ so as to represent the physically meaningful interactions of the system. However, only two IDPs^40,41^ were studied and the general applicability of this approach has not been established.

Here, we report that HREMD produces configurational ensembles consistent with SAXS, SANS, and NMR experiments for three IDPs with markedly different sequence characteristics: Histatin 5 (24 residues) and Sic 1 (92 residues), both of which have an abundance of positively charged residues, and the N-terminal SH4UD (95 residues) of c-Src kinase, which contains positively and negatively charged residues mixed over the sequence. The HREMD results are in agreement with experimental data on both local and global properties when employing either of two force fields (Amber ff03ws^20^ with TIP4P/2005s^20^ and Amber ff99SB-*disp*^17^ with modified TIP4P-D^17^, hereafter termed as a03ws and a99SB-disp, respectively). In contrast, standard MD simulations of equivalent computational cost as HREMD generate ensembles consistent only with NMR chemical shifts, but not with SAXS. Further, the HREMD ensembles of IDPs are found to be described by a theoretical semiflexible polymer chain model quantifying the stiffness and strength of interaction with the solvent. We suggest “best practices” in achieving accurate and efficient IDP sampling using HREMD and discuss differences in the size between Sic 1 and SH4UD. The results demonstrate quite clearly that the recently optimized force fields are reliable and that the current major impediment to accurate simulation of IDPs is sampling. HREMD is therefore the present tool of choice for obtaining atomic-detailed IDP ensembles.

## Results

### HREMD ensembles in agreement with SAXS, SANS, and NMR

**Fig. 1:**
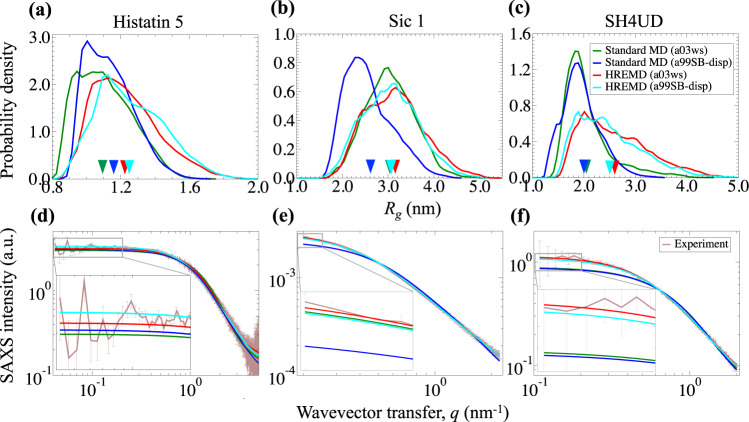
Comparison of experiemntal and calculated global structural properties of IDPs. **a**–**c** The histograms of Rg of **a** Histatin 5, **b** Sic 1, and **c** SH4UD obtained from MD simulations. The inverted triangles indicate the average *R*_g_ of each simulation. **d**–**f** The SAXS profiles calculated from simulations (using SWAXS^46^) are compared to experiments for **d** Histatin 5^31^, **e** Sic 1^47^, and **f** SH4UD^41^. Insets: SAXS data are zoomed at low-*q* values to show the differences in intensity for different force fields and sampling methods. In all cases, the color code indicates the force fields, a03ws^20^ or a99SB-disp^17^, and sampling methods, standard MD or HREMD (Supplementary Tables 1 and 2). HREMD results are from the lowest rank replica of the simulations shown by the bold-italics font in Supplementary Table 2. SANS data of SH4UD are shown in Supplementary Fig. 2.

We conducted HREMD simulations of three IDPs with varying amino acid composition (Supplementary Note and Supplementary Fig. 1), employing two force fields: a03ws^20^ and a99SB-disp^17^. Each replica of HREMD simulation is 500 ns long (Supplementary Tables 1–4). For comparison, we also conducted standard MD, that is, without enhancing the sampling, of the same cumulative length as the HREMD (Supplementary Tables 1–4). The cumulative lengths of standard MD simulations for Histatin 5, Sic 1, and SH4UD are 5, 8, and 10 μs, respectively. The histograms of a radius of gyration (*R*_g_) show the IDPs adopt a continuum of collapsed to extended structures (Fig. 1a–c).

The global, ensemble-averaged properties of IDPs such as *R*_g_, shape, and chain statistics can be derived using small-angle scattering. We calculated the ensemble-averaged theoretical SAXS and SANS curves from the simulation trajectories, by taking into account explicitly the protein hydration shell and without reweighting, and compared them directly to the experiments. We found an excellent agreement of the HREMD-generated ensembles with SAXS and SANS measurements for both force fields (SAXS in Fig. 1d–f and SANS in Supplementary Fig. 2), whereas the standard MD simulations were found to deviate from the experiments, except for Sic 1 with a03ws. The agreement between simulation and experiment was quantified by the *χ*^2^ value as defined in Eq. (5) and listed in Supplementary Table 5. *χ*^2^ calculated from HREMD converges in ~100 ns for Histatin 5, but in ~300–400 ns for the larger Sic 1 and SH4UD (Supplementary Fig. 3).

The histograms of *R*_g_ show that standard MD simulations sample more compact structures than does HREMD with the same force fields. Moreover, the histograms of *R*_g_ from all the independent standard MD runs are different from each other (Supplementary Fig. 4), suggesting lack of convergence between the MD runs due to inadequate sampling. Therefore, for the IDPs studied here, poor agreement with experiment arises primarily from insufficient sampling rather than from shortcomings of the force fields.

**Fig. 2:**
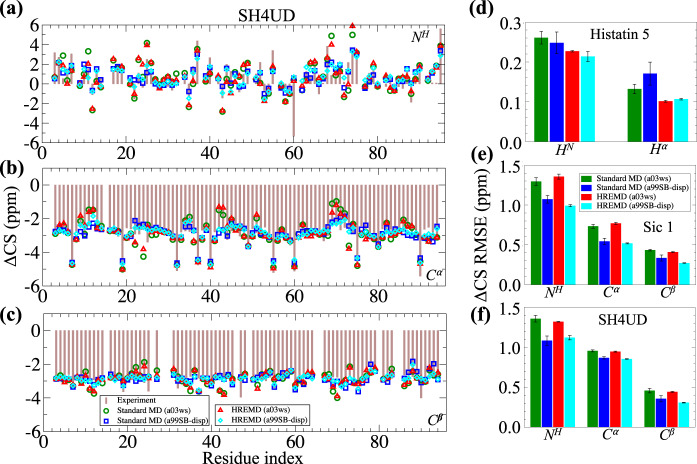
Comparison of experimental and calculated local structural properties of IDPs. Comparison between the ensemble-averaged experimental (bars) and calculated (symbols) NMR secondary chemical shifts (ΔCS) of backbone atoms **a** N^H^, **b** C^α^, and **c** C^β^ for SH4UD. ΔCS RMSE of backbone atoms with respect to experimental values (bars), as defined in Eq. (6), for **d** Histatin 5^49^, **e** Sic 1^47^, and **f** SH4UD^50^. The error bars in ΔCS RMSE (**d**–**f**) are the standard error of the mean as defined in Eq. (4). The color code indicates the force field and sampling method used. The theoretical NMR chemical shifts are calculated using SHIFTX2^48^. The prediction values of SHIFTX2 have RMS errors of 1.12, 0.44, 0.52, 0.17, and 0.12 p.p.m. for backbone atoms N^H^, C^α^, C^β^, H^N^, and H^α^, respectively^48^.

NMR chemical shifts (CS) provide information on the local chemical environment of protein atoms and reflect structural factors such as backbone and side-chain conformations. The ensemble-averaged NMR secondary CS (ΔCS) calculated from all the simulations (force fields and sampling methods) are in a good agreement with the experimental values (Fig. 2a–c and Supplementary Figs. 5–8). The quality of agreement is determined from the root mean square (RMS) error defined in Eq. (6) for each backbone atom (Fig. 2d–f), which is of the order of predicted RMS errors of SHIFTX2^48^ (viz, 1.12, 0.44, 0.52, 0.17, and 0.12 p.p.m. for N^H^, C^α^, C^β^, H^N^, and H^α^ atoms, respectively^48^). However, a slightly better agreement between calculated and experimental ΔCS was obtained using a99SB-disp (Fig. 2d–f). Importantly, the agreement was not improved by enhancing the sampling by HREMD. Moreover, we found that the standard MD ensembles are consistent with NMR CS but not with small-angle scattering experiments. This indicates that CS alone may be an insufficient criterion to test the validity of IDP ensembles.

Both force fields and sampling methods predict nearly the same transient secondary structure elements. Transient helices, which are considered to be biologically relevant^51–53^, were found proximal to known phosphorylation residues of Sic 1^47^ and to known lipid-binding or phosphorylation residues in SH4UD^50,54^. In contrast, the propensity of each secondary structure element is found to depend on both the force fields and sampling methods (Supplementary Figs. 9 and 10). The IDPs we studied mostly showed a high propensity for coils that lack secondary structure, consistent with the lack of long-range contacts found in the simulations (Supplementary Fig. 11).

### Polymer properties

**Fig. 3:**
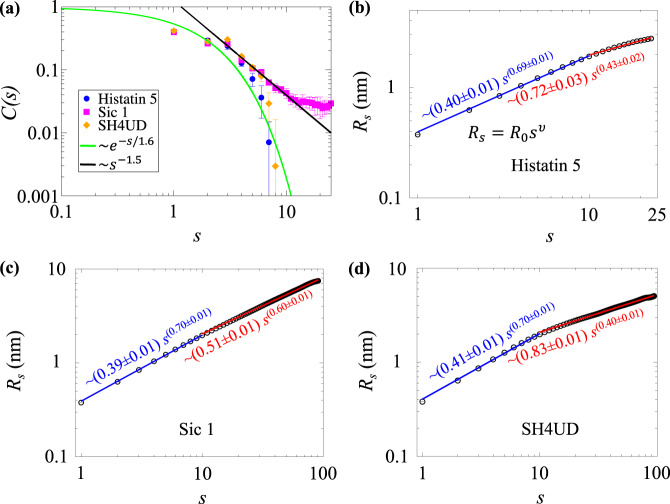
Chain statistics of IDPs. **a** The orientational correlation function as a function of the pairwise residue sequence separation, *s*. For $s \,< \,5$, $C\left( s \right)$ is fitted by $C\left( s \right) = e^{ - s/k}$ for each IDP, where *k* is the number of C_α_ atom pair related to persistence length (*l*_p_) by *l*_p_ = *k* × 0.38 nm. For $s \ge 5$ the power law $C\left( s \right)\sim s^{ - 3/2}$ applies only for Sic 1, whereas for Histatin 5 and SH4UD the correlation vanishes. **b**–**d** The average pairwise geometric distance (*R*_*s*_) between C_α_ atoms of two residues at separation *s* for **b** Histatin 5, **c** Sic 1, and **d** SH4UD. The data are fitted by Eq. (2) in two regimes, *s* ≤ 10 (blue) and *s* > 10 (red). The error bars are smaller than the symbol size.

We estimated the stiffness of the protein backbones by calculating the orientational correlation function

1 $$C\left( s \right) = < {\boldsymbol{n}}_{\boldsymbol{i}} \cdot {\boldsymbol{n}}_{{\boldsymbol{i}} + {\boldsymbol{s}}} > $$

where $s = |i - j|$ is the pairwise residue separation (sometimes called contour length), and ${\boldsymbol{n}}_{\boldsymbol{i}}$ is the unit vector connecting C_α_ atoms of two consecutive residues (Fig. 3a). The steeper the decay of *C*(*s*), the lower the stiffness of the chain. *C*(*s*) is similar for the three IDPs for *s* < 5, exhibiting an exponential decay $C\left( s \right) = e^{ - s/k}$, where *k* is the number of C_α_ atom pairs corresponding to the persistence length (*l*_p_). *l*_p_ provides the maximum size of a protein segment over which the structural fluctuations are correlated. In other words, it is the measure of stiffness of a polypeptide chain. Here, we approximate *l*_p_ = *k* × 0.38 nm, where 0.38 nm is the distance between two consecutive C_α_ atoms in proteins^55^. We obtain *k* = 1.6 and *l*_p_ = 0.61 ± 0.02 nm for all IDPs, in close agreement to a value of *l*_p_ = 0.40 ± 0.07 nm reported for unfolded (hCyp, CspTm, R15, and R17) and disordered (IN and ProTα, variants ProT53 and ProT54) proteins^55^. A power-law decay (*~s*^−3/2^) is found for Sic 1 at 5 < *s* ≤ 13, whereas correlations decay more rapidly and vanish for *s* > 5 for Histatin 5 and SH4UD. Therefore, Sic 1 is the stiffest.

The statistics of internal distances (“scaling properties”) of polymers in dilute solution can be characterized using the Flory scaling law given by Eq. (2):

2 $$R_s = R_0s^\upsilon$$

where $R_s$ is the average intraprotein pairwise distance between the C_α_ atoms of residues *i* and *j* at separation *s* = |*i* *−* *j*|, the prefactor $R_0$ is a constant and *ν* is the Flory exponent. Balanced polymer-solvent and intrapolymer interactions give rise to Gaussian coil and *ν* = 0.5, while a self-avoiding random walk with *ν* = 0.588 is predicted when the polymer–water interactions are favored. Interestingly, we found two different power-law regimes are needed to fit the data^56,57^ (Fig. 3b–d). At short contour lengths (*s* ≤ 10), all three IDPs show common behavior. Although the scaling law (Eq. (2)) is formally valid only for large chain lengths *s*, we fit the data for *s* < 10 with Eq. (2) to demonstrate that the IDPs have similar local chain statistics, evidenced by similar fits with $\nu$ ≈ 0.70 and prefactor of *R*_0_ ~ 0.4 nm (*R*_0_ is similar to the average distance between two consecutive C_α_ atoms). On the other hand, at longer residue separations (*s* > 10) the *R*_*s*_ of the three IDPs deviate. Histatin 5 and SH4UD with $\nu$ ≈ 0.43 and 0.40, respectively, adopt more collapsed global conformations than self-avoiding random walk. In contrast, Sic 1 ($\nu$ ≈ 0.60) remains stiff even at longer residue separations.

## Discussion

IDPs present a new paradigm for understanding flexibility–function relationships in biology^1,58–60^. Currently, it is not possible to determine the ensemble of the 3D structures that an IDP adopts from either experiment or simulation alone. The number of experimental observables is considerably smaller than the number of the IDP’s configurational degrees of freedom, making model reconstruction from experimental data a highly underdetermined problem. For MD simulations, although improved molecular mechanics methods perform well for small model disordered peptides^2,19,29,31,32^, it has been necessary to bias or reweight the MD results to achieve consistency with experiments^35,36,38,39,61–63^. The reason MD has not always been accurate was unclear: it could have been deficiencies in the force fields, insufficient sampling, or both.

Here, we demonstrated that HREMD reproduces key experimental observables (SAXS, SANS, and NMR) using two different force fields for three different IDPs. In contrast, the ensemble generated by standard MD of equivalent length failed to match SAXS data (Fig. 1). The comparison of standard MD and HREMD using the same force field suggests that the a03ws and a99SB-disp force fields are of adequate accuracy and that enhanced sampling techniques are necessary to reproduce the experimental data.

We found that the calculated NMR CS and the loci of secondary structure elements are the easiest to converge as they are consistent between all the simulations, independent of force field and sampling method. In contrast, HREMD is required for SAXS observables to converge to the experimental values. The most difficult quantities to converge are the secondary structure propensities, which were found here to depend on both the force field and the sampling method, perhaps more on the former than the latter (Supplementary Figs. 9 and 10), with a03ws and a99SB-disp having biases towards helices and β-sheets, respectively.

The data show that standard MD simulations can be in apparent agreement with NMR CS, which measure local structural information^64^, while failing to reproduce SAXS/SANS intensities, which determine with high precision more global structural properties (here distributions of distances between pairs of nuclei that are >~1 nm apart^61,65^) (Figs. 1 and 2 and Supplementary Fig. 2)^41^. Thus, agreement with NMR CS alone is not always a definitive test of the accuracy of MD simulations of IDPs. It is critical to analyze and compare both local and global properties^17,66^ of IDPs to ensure that the simulations have indeed generated accurate ensembles.

Simple theories established for semiflexible homopolymers and heteropolymers have been shown to provide a qualitative description of IDP structural properties such as stiffness^67–69^ and solvent quality^12,14,55,70,71^. The high fidelity HREMD trajectories reveal that, despite having markedly different sequences, the IDPs studied here have common hierarchical chain architecture. For short contour lengths (up to ~10 residues), the chain statistics of all three IDPs are similar, as evidenced by *R*_*s*_ and *C*(*s*). These short segments are relatively stiff. Beyond this critical contour length, the IDPs differentiate. SH4UD and Histatin 5 become flexible, while Sic 1 remains relatively stiff with power-law decay in *C*(*s*) that implies long-range spatial correlations^68^. This is consistent with Sic 1 being more extended than SH4UD.

The origin of the stiffness of Sic 1 relative to SH4UD can be understood by examining their primary sequences (Supplementary Fig. 1). All the charged residues of Sic 1 are positive, leading to electrostatic repulsion between them. Further, Sic 1 contains 15 proline and 5 glycine residues. Proline is stiff due to its cyclic side chain, whereas the absence of a side chain for glycine increases the backbone flexibility^72,73^, and therefore both are known to be disorder-promoting^72,73^. In comparison, SH4UD has both positively and negatively charged residues, 11 prolines and 12 glycines.

We now discuss the HREMD method^42,43,45^ and make recommendations for its optimal use in IDPs. HREMD enhances sampling by changing the quality of water as a good solvent for an IDP. This is achieved by scaling only the intraprotein and protein-solvent potential energy functions by a factor, $\lambda$ and $\sqrt \lambda$, respectively (where $\lambda$ < 1). An exchange of coordinates is allowed between neighboring replicas if the Monte Carlo metropolis criterion is satisfied^42,43^. The HREMD method was chosen because it does not necessitate a predefined reaction coordinate. The advantage of HREMD over temperature replica exchange MD is that HREMD crosses entropic barriers^74^ more efficiently and a smaller number of replicas is sufficient, that is, HREMD is computationally more efficient.

The total number of replicas (*n*) used, the scaling factor (*λ*_*i*_), or the effective temperature (*T*_*i*_) of a replica and the average exchange probability (*p*_ex_) of the lowest rank replica are listed in Supplementary Tables 2 and 3. A *T*_max_ of 400–450 K (lower limit) was needed to achieve a good agreement between the lowest rank (unscaled) replica of HREMD and the experimental SAXS results. Moreover, to estimate the upper limit of effective temperature, we performed HREMD of Histatin 5 using a99SB-disp, *T*_max_ = 800 K and 24 replicas (Supplementary Table 3). This simulation generated the ensemble in the lowest rank replica similar to that of HREMD with *T*_max_ = 450 K (Supplementary Fig. 12a). However, we noted that replica from *T*_*i*_ = 522 K and above-sampled collapsed structures when compared to the ensemble of the lowest rank replica. Therefore, we suggest 450 K < *T*_max_ < 500 K is an appropriate choice for the upper limit of maximum effective temperature (Supplementary Fig. 12a). However, choosing the higher value of *T*_max_ would increase the number of replicas and thus computational cost.

To ensure HREMD does not bias the ensemble, we also performed control simulations of a short intrinsically disordered peptide, Ala_5_ (five residues)^17,20^ and the 20-residue folded protein Trp cage^17,75^ (Supplementary Fig. 13), both of which have been used as benchmarks for the optimization of molecular mechanics force fields^17,20^. Unlike what was observed for the longer IDPs (Histatin 5, Sic 1, and SH4UD), MD, and HREMD both yield similar ensembles for the controls (Supplementary Fig. 13). This suggests that HREMD does not introduce unphysical conformations and is equivalent to microsecond standard MD for short peptides and proteins.

A quantitative comparison of the sampling efficiency of standard MD and HREMD is provided by calculating the autocorrelation functions (*C*_t_) of the number of contacts (*n*_c_) and of *R*_g_ (Supplementary Note, Supplementary Fig. 14, and Supplementary Table 6). The decay of the autocorrelation is markedly more pronounced for HREMD than for standard MD. Taking the steepness of the decay of *C*_t_ in Supplementary Fig. 14 as a measure of sampling efficiency, it is clear that HREMD sampling is superior to that of standard MD (Supplementary Table 6).

In summary, we demonstrate HREMD simulations as an effective method to generate accurate structural ensembles of three IDPs with varying amino acid composition (Histatin 5, Sic 1, and SH4UD). The unbiased HREMD trajectories, calculated without using any experimental input or predefined reaction coordinate, are in excellent agreement with SAXS, SANS, and NMR observables. Nonetheless, comparison to experimental data was imperative to confirm the accuracy of MD results. The success of the HREMD approach for these three markedly different IDPs suggests that it will be of general applicability. Moreover, HREMD simulations performed using two recent molecular mechanics force fields (a03ws and a99SB-disp) converge to the same distribution of *R*_g_. In contrast, neither of the force fields could reproduce small-angle scattering experiments with standard MD of the same cumulative length as HREMD, although NMR CS were reproduced accurately with standard MD. Local chemical and structural properties of IDPs, which influence CS, therefore, seem force field-dependent, while the overall protein size and shape, which influences small-angle scattering intensities, also depend on the sampling. Both local and global features must be employed to validate IDP ensembles. Therefore, our results suggest adequately sampled simulations using recent IDP-specific force fields can reliably generate the 3D ensembles of IDPs (Supplementary Fig. 15), which is a prerequisite to an understanding of the biological function of IDPs. We also report that despite differences in their sequence, all three IDPs have similar local chain statistics for short lengths (<~10 residues). More studies are required to establish whether this is a universal IDP behavior.

## Methods

### Experimental SAXS and NMR data

The experimental SAXS data of Histatin 5, Sic 1, and SH4UD were taken from Henriques et al.^31^ Protein Ensemble Database (http://pedb.vib.be)^47^ and our previous work^41^, respectively. Similarly, NMR CS of backbone atoms (C^α^, C^β^, N^H^, H^α^, and H^N^) of Histatin 5, Sic 1, and SH4UD were acquired from the literature^49^, Protein Ensemble Database^47^, and Biological Magnetic Resonance Data Bank database entry 15563^50^ respectively.

### MD simulations

The initial 3D structures of IDPs (Histatin 5, Sic 1, and SH4UD) were obtained from I-TASSER^76^. An MD-equilibrated starting structure with *R*_g_ value close to experimental SAXS was chosen for the production simulation of each IDP. The same starting structure of IDP was utilized for each force field and sampling method. A short disordered peptide, Ala_5_^17,20^, and a small folded protein, Trp-cage^17^, were also simulated as controls (SI). The initial structure of Ala_5_ was constructed using Visual MDs^77^, whereas the starting structure of Trp cage was taken from PDB 1L2Y^75^.

We performed standard MD simulations with two recently optimized force fields, Amber ff03ws^20,78,79^ with TIP4P/2005s^20^ (a03ws) and Amber ff99SB-*disp*^17,80^ with the modified TIP4P-D^17,22^ water model (a99SB-disp) using GROMACS^81–86^. All bonds involving hydrogen atoms were constrained using the LINCS algorithm^87^. The SETTLE algorithm was used for water^88^. The Verlet leapfrog algorithm was used to numerically integrate the equation of motions with a time step of 2 fs. A cutoff of 1.2 nm was used for short-range electrostatic and Lennard–Jones interactions. Long-range electrostatic interactions were calculated by particle-mesh Ewald^89^ summation with a fourth-order interpolation and a grid spacing of 0.16 nm. The solute and solvent were coupled separately to a temperature bath of 300, 293, 300, 298, and 282 K for Histatin 5, Sic 1, SH4UD, Ala_5_, and Trp cage, respectively, to match the temperatures measured at the experiments using velocity-rescaling thermostat^90^ with a relaxation time of 0.1 ps. The pressure coupling was fixed at 1 bar using the Parrinello–Rahman algorithm^91^ with a relaxation time of 2 ps and isothermal compressibility of 4.5 × 10^−5^ bar^−1^. The energy of each system was minimized using 1000 steepest decent steps followed by 1 ns equilibration at NVT (amount of substance, volume, and temperature) and NPT (amount of substance, pressure, and temperature) ensembles. The production runs were carried out in the NPT ensemble. The cumulative lengths of standard MD simulations with a number of independent runs enclosed in the brackets for Ala_5_, Trp cage, Histatin 5, Sic 1, and SH4UD are 2 (1), 4 (4), 5 (5), 8 (4), and 10 μs (6), respectively (Supplementary Table 1).

### Enhanced sampling MD simulations

We employed replica exchange with solute tempering 2^42,43^, an HREMD simulation method to enhance the conformational sampling. Replica exchange with solute tempering 2 is implemented in GROMACS (v.2018.6)^81–86^ patched with PLUMED (v.2.5.2)^92^. The interaction potentials of intraprotein and protein solvent were scaled by a factor *λ* and *√λ*, respectively, while water–water interactions were unaltered^42–44,93^. The scaling factor *λ*_*i*_, and corresponding effective temperatures *T*_*i*_ of the *i*th replica are given by,

3 $$\lambda _i = \frac{{T_0}}{{T_i}} = {\mathrm{exp}}\left( { - \frac{i}{{(n - 1)}}{\mathrm{ln}}\left( {\frac{{T_{\mathrm{max}}}}{{T_0}}} \right)} \right)$$

where *T*_0_ and *T*_max_ are the effective temperatures of lowest rank (unscaled) and the highest rank replicas, respectively, and *n* is the total number of replicas used. For analysis, we use only the trajectory of the unscaled for lowest rank replica (*λ*_0_ = 1 or *T*_0_). Exchange of coordinate between neighboring replicas was attempted every 400 MD steps. Each replica of HREMD is 500 ns long. The cumulative lengths of HREMD simulations with the number of replicas enclosed in the brackets for Ala_5_, Trp-cage, Histatin 5, Sic 1, and SH4UD are 2 (4), 4 (8), 5 (10), 8 (16), and 10 μs (20), respectively (Supplementary Tables 2 and 3). The details of HREMD and standard MD simulations are shown in Supplementary Tables 1–4. The secondary structure prediction was calculated with DSSP^94^. The orientational correlation function is determined using MD analysis^95^.

### Statistics and reproducibility

To estimate the error from HREMD trajectory, we divided the trajectory into five equal blocks each containing 10,000 frames (0–100, 100–200, 200–300, 300–400, and 400–500 ns). The mean value for each block, *m*_*i*_ (*i* = 1–5), was first calculated. The reported error bars are the standard error of the mean of the (*m*_1_, *m*_2_, *m*_*3*_, *m*_4_, and m_5_) distribution, that is,

4 $${\mathrm{Error}}\,{\mathrm{bar}} = \sqrt {\frac{1}{{n(n - 1)}}\mathop {\sum }\limits_i^{n = 5} \left( {m_i - {\overline{m}}} \right)^2}$$

where $\overline m$ is the mean value and *n* = 5 is the number of blocks used.

In regard to the reproducibility of the work, a multiple copies of standard MD and two copies of HREMD simulations were performed for each IDP.

### Theoretical SAXS profiles

The theoretical SAXS and SANS intensities were calculated with SWAXS^46,96^ and SASSENA^97^, respectively, by taking into account of explicit hydration water, which contributes to the signal^46^. The agreement between experiment and simulation was determined by a *χ*^2^ value:

5 $$\chi ^2 = \frac{1}{{k - 1}}\mathop {\sum }\limits_{i = 1}^k \left. {\left\{ {\frac{{[ < I_{\mathrm{expt}}\left( {q_i} \right) > - (c \,< \,I_{\mathrm{sim}}\left( {q_i} \right) > + {\mathrm{bgd}})]}}{{\sigma _{\mathrm{expt}}(q_i)}}} \right.} \right\}^2$$

where <*I*_expt_(*q*)> and <*I*_sim_(*q*)> are the ensemble-averaged experimental and theoretical SAXS data, respectively, *k* is the number of experimental *q* points, *c* is a scaling factor, bgd is a constant background, and *σ*_expt_ is the experimental error. In Eq. (5), *c* is a factor to scale calculated values to the experiment because the experimental values are often expressed in arbitrary units. It does not change the shape of the SAXS curve. Similarly, bgd is used to incorporate the uncertainty due to mismatch in buffer subtraction at higher *q* values^14^ in the experiment.

### Theoretical NMR CS

The theoretical NMR CS was calculated with SHIFTX2^48^ by taking the average over all frames from the MD trajectory. Furthermore, we determined the NMR secondary CS for N^H^, C^α^, C^β^, H^N^, and H^α^ atoms as the difference between the experimental (or simulation-derived) CS and the corresponding random coil values specific to a particular atom and amino acid:

$$\Delta {\mathrm{CS}}^{\mathrm{expt}}\left( {x,i} \right) = {\mathrm{C}S}^{\mathrm{expt}}\left( {x,i} \right) - {\mathrm{CS}}^{\mathrm{RC}}(x,i)$$

$$\Delta {\mathrm{CS}}^{\mathrm{calc}}\left( {x,i} \right) = {\mathrm{CS}}^{\mathrm{calc}}\left( {x,i} \right) - {\mathrm{CS}}^{\mathrm{RC}}(x,i)$$

where atom *x* ∈ N^H^, C^α^, C^β^, H^N^, and H^α^ and *i* refers to a specific amino acid. ${\mathrm{CS}}^{\mathrm{expt}}\left( {x,i} \right)$, ${\mathrm{CS}}^{\mathrm{calc}}(x,i)$, and ${\mathrm{CS}}^{\mathrm{RC}}(x,i)$ are the CS values from experiment, MD (calculated) and random coil database for atom “*x*” and amino acid “*i*,” respectively. Note that the calculated CS for each atom are corrected as ${\mathrm{CS}}^{\mathrm{calc}} = {\mathrm{CS}}^{{\mathrm{calc}}0} + O$, where ${\mathrm{CS}}^{{\mathrm{calc}}0}$ is an actual ensemble-averaged value from SHIFTX2 and *O* is an offset constant determined from linear regression fit of the theoretical to the experimental NMR CS (Supplementary Figs. 5–7). Such an offset may arise from the systematic or referencing error in the NMR CS measurement or calculation^98^ and is used here to improve the agreement between experiment and calculated values. ${\mathrm{CS}}^{\mathrm{RC}}$ values are those reported in Tamiola et al.^99^. Finally, we quantified the agreement between experimental and calculated secondary CS of atom by evaluating the RMS error given by,

6 $${\mathrm{RMSE}}(x) = \sqrt {\frac{1}{n}\mathop {\sum }\limits_{i = 1}^n (\Delta {\mathrm{CS}}^{\mathrm{expt}}\left( {x,i} \right) - \Delta {\mathrm{CS}}^{\mathrm{calc}}\left( {x,i} \right))^2}$$

where *n* is the total number of residues in the IDP.

### Reporting summary

Further information on research design is available in the Nature Research Reporting Summary linked to this article.

## Supplementary information

Supplementary Information

Description of Supplementary Files

Supplementary Data 1

Supplementary Data 2

Supplementary Data 3

Supplementary Data 4

Reporting Summary

## Data Availability

The datasets generated during and/or analyzed during the current study are available from the corresponding author on reasonable request. The source data for the Figs. 1, 2, and 3 are available as Supplementary Data 1, Supplementary Data 2, and Supplementary Data 3, respectively.

## Code availability

The input files for running HREMD simulation using GROMACS patched with PLUMED are provided as Supplementary Data 4. It is also deposited in Zenodo^100^ and GitHub (https://github.com/utsabstha/hremd-idp).
