# Supplementary material for: Full structural ensembles of intrinsically disordered proteins from unbiased molecular dynamics simulations
Source: Commun Biol. 2021 Feb 23;4:243. doi: 10.1038/s42003-021-01759-1 (PMC7902620; doi:10.1038/s42003-021-01759-1)
Supplement: Supplementary file 1 — Supplementary Information [file 42003_2021_1759_MOESM1_ESM.pdf]

Supplementary Information

Full structural ensembles of intrinsically disordered proteins from unbiased molecular dynamics simulations

Utsab R. Shrestha[1], Jeremy C. Smith[1,2], Loukas Petridis[1,2*]

[1]UT/ORNL Center for Molecular Biophysics, Biosciences Division, Oak Ridge National Laboratory, Oak Ridge, TN 37831, United States.
[2]Department of Biochemistry and Cellular and Molecular Biology, University of Tennessee, Knoxville, TN 37996, United States.

[*]Corresponding author: petridisl@ornl.gov

Supplementary Note.

Sequence and chemical properties of IDPs.

The sequence of Histatin 5, Sic 1 and SH4UD are shown in **Supplementary Fig. 1a**. The net charge per residue (NCPR) of each protein sequence was calculated as follows,

$$NCPR = \frac{1}{n}\left|\sum_{i=1}^{n} q_i\right| \quad \text{(S1)}$$

where q_i is the charge on i^{th} residue and, n is the total number of residues and |…| represents the absolute value. ExPASy[1] was used to calculate the normalized hydrophobicity of each protein residue by the Kyte and Doolittle approximation[2] with a window size of 5 residues and normalized to a scale of 0 to 1. The mean normalized hydrophobicity (MNH) is defined as,

$$MNH = \frac{1}{n}\sum_{i=1}^{n} H_i^{norm} \quad \text{(S2)}$$

where H_i^{norm} is the normalized hydrophobicity of residue i to a scale of 0 to 1 of each amino acid residue and n is the total number of residues in a protein sequence. NCPR vs. MNH of each protein sequence is plotted in **Supplementary Fig. 1b**. Sic 1 and SH4UD have nearly the same number of amino acid residues and MNH, but different NCPR.

MD simulation details.

The standard MD and HREMD simulations were conducted using two force fields, Amber ff03ws with TIP4P/2005s[3] water model (a03ws) and Amber ff99SB-*disp* with the TIP4P-D[4] water model (a99SB-disp).The details of the simulations are shown in the **Supplementary Tables 1-4** below.

Quality of agreement between small-angle scattering experiments and MD simulations.

The quality of agreement of theoretical SAXS and SANS profiles to experimental data is quantified by calculating chi-square (χ^2) using **Eq. (5)**. The values of χ^2 for IDPs are listed in **Supplementary Table 5**.

Comparison of experimental and calculated NMR chemical shifts of backbone atoms in IDPs.

The linear regression analysis is shown to compare the agreement between experimental and MD-calculated chemical shifts of IDPs. The *offset* values obtained from linear fit are used in $CS^{calc} = CS^{calc0} + offset$, where CS^{calc0} is an actual ensemble-averaged value from SHIFTX2. Such *offset* may arise from the systematic or referencing error in the NMR chemical shift measurement or calculation[6] and used to improve the agreement between experiment and calculated values.

Propensity of coil and secondary structures in IDPs.

Histatin 5, Sic 1 and SH4UD are shown to possess transient 3_{10}- and α- helices, but negligible β-sheets (**Supplementary Figs. 8 and 9**). Moreover, the inter-residue contact maps show a lack of long-range interaction in IDPs (**Supplementary Fig. 10**).

Autocorrelation of inter-residue contact and R_g.

To illustrate the efficiency of sampling, we calculated the number of inter-residue contacts, n_c. A contact is defined here when any heavy atom in residue i is at a distance less than 0.45 nm from any heavy atom in residue j with $|i\text{-}j|>3$. We compare the autocorrelation function, C_t of the n_c (**Supplementary Fig. 14a-c**) and R_g (**Supplementary Fig. 14d-f**) for the standard MD with

those from the HREMD. Taking the steepness of the decay of C_t as a measure of sampling efficiency, it is clear that HREMD is superior to standard MD.

C_t for standard MD simulations was fitted with the sum of two exponential functions given by,

$$C_t = a_1 e^{-t/\tau_1} + a_2 e^{-t/\tau_2}$$

where τ_1 and τ_2 are the correlation time constants for faster and slower decay processes, respectively. The estimation of τ_2 has high uncertainty due to noisy data at longer timescales, even though a small error is obtained from the fit of exponential function. We found the values of τ_1 and τ_2 are of the order of ~1-10 and 10-100 ns respectively. Correlation times were shorted for the smaller Histatin 5. The HREMD autocorrelation decays steeply, and thus could not be fitted with exponential decay function. For quantitative comparison between standard MD and HREMD, we crudely estimated the correlation time τ, such that $C_t(t=\tau)=e^{-1}$. The autocorrelation times are at least 1000 larger for standard MD than HREMD (**Supplementary Table 6**). By this crude metric, HREMD sampled conformational states about three orders of magnitude faster than did conventional MD.

Amino acid sequence of IDPs

(a)

```
>Histatin 5 (24 residues)
DSHAKRHHGY 10 KRKFHEKHHS 20 HRGY

>Sic 1 (92 residues)
GSMTPSTPPR 10 SRGTRYLAQP 20 SGNTSSSALM 30 QGQKTPQKPS 40 QNLVPVTPST 50
TKSFKNAPLL 60 APPNSNMGMT 70 SPFNGLTSPQ 80 RSPFPKSSVK 90 RT

>SH4UD (95 residues)
MGSNKSKPKD 10 ASQRRRSLEP 20 AENVHGAGGG 30 AFPASQTPSK 40 PASADGHRGP 50
SAAFAPAAAE 60 PKLFGGFNSS 70 DTVTSPQRAG 80 PLAGGSAWSH 90 PQFEK
```

Red: Positively charged
Magenta: Negatively charged
Green: Polar (neutral)
Blue: Hydrophobic

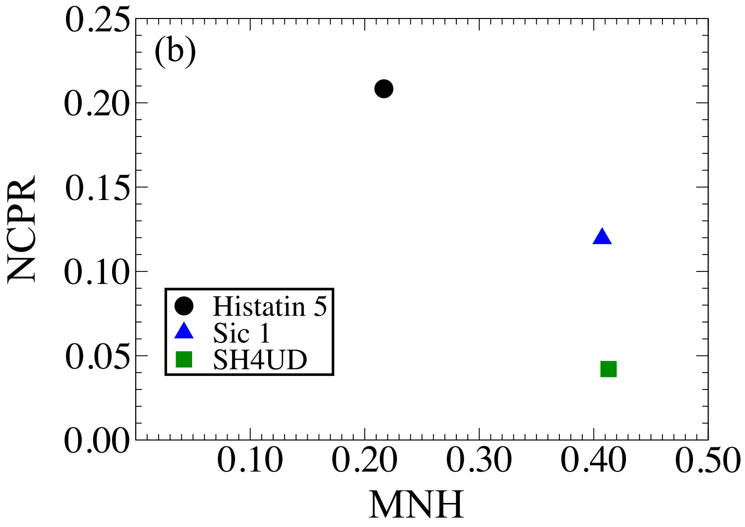

Supplementary Fig. 1. (a) Sequence of the three IDPs (Histatin 5, Sic 1 and SH4UD) studied here. The red, magenta, green and blue colored letters represent positively charged, negatively charged, neutral polar and hydrophobic residues respectively. (b) Net charge per residue (NCPR) vs. mean normalized hydrophobicity (MNH) plot, also known as Uversky diagram is shown.

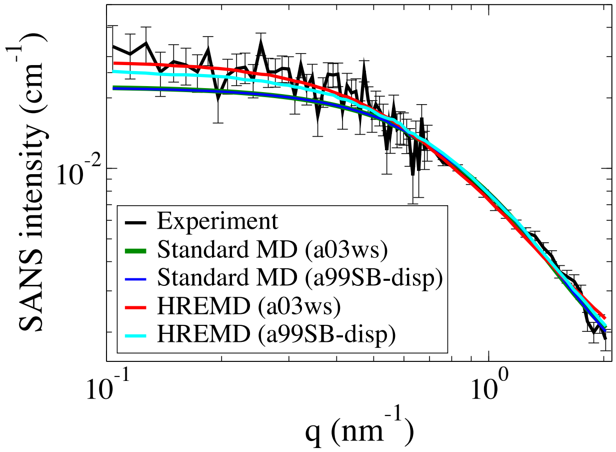

Supplementary Fig. 2. Experimental (black) and simulation-derived small-angle neutron scattering (SANS) profiles of SH4UD are compared. The theoretical SANS data are calculated taking into account of explicit hydration water around SH4UD using software SASSENA[5] from standard MD and HREMD simulations of a03ws and a99SB-disp force fields. Note: the green and blue curves overlap.

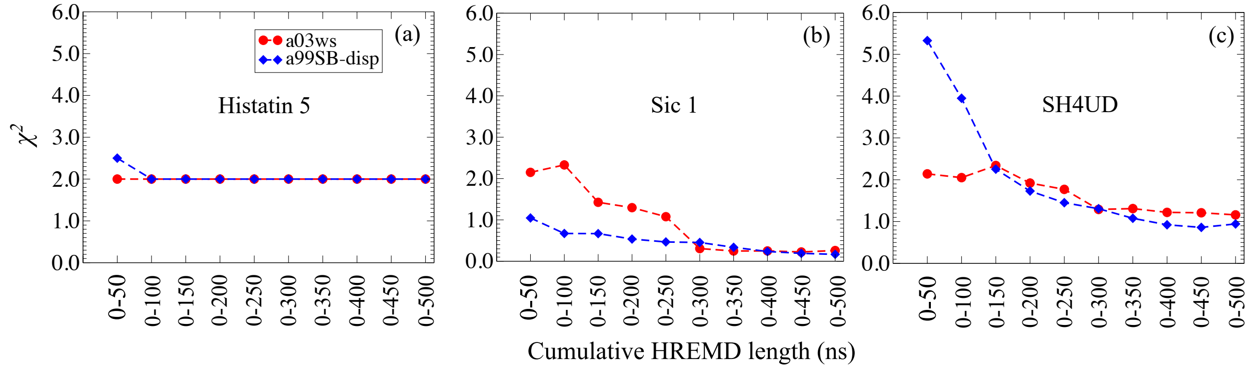

Supplementary Fig. 3. χ^2 values quantifying the agreement between theoretical vs. experimental SAXS data (**Eq. 5**) with respect to cumulative length of HREMD simulations.

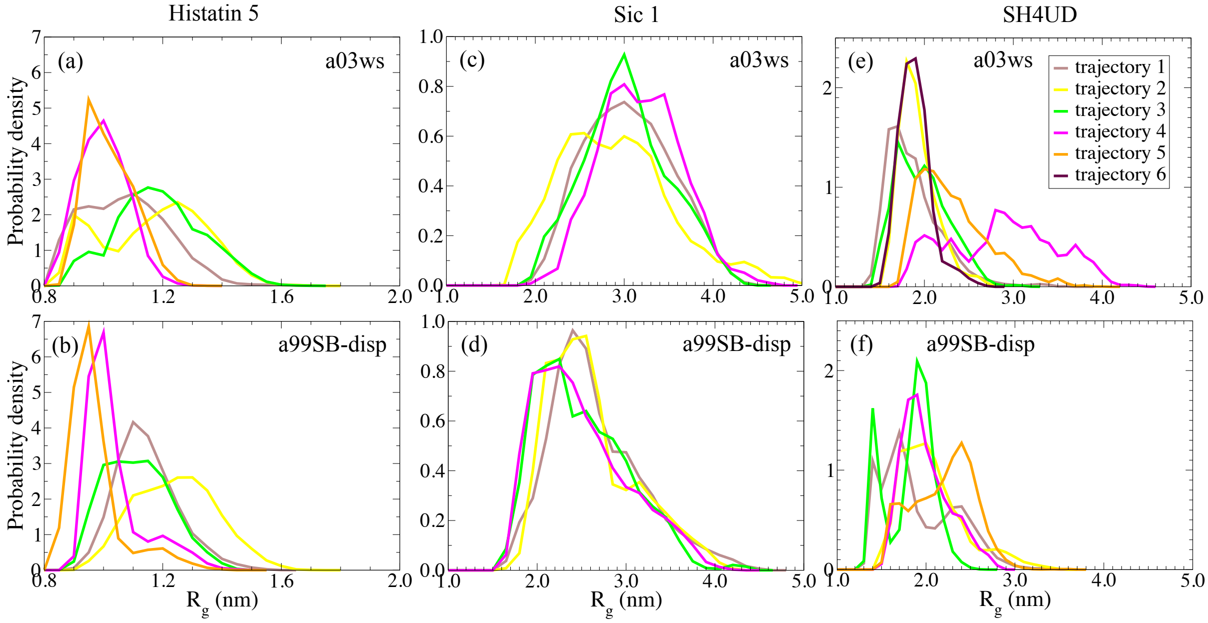

Supplementary Fig. 4. The histograms of R_g of (a, b) Histatin 5, (c, d) Sic 1 and (e, f) SH4UD obtained from the individual standard MD simulations.

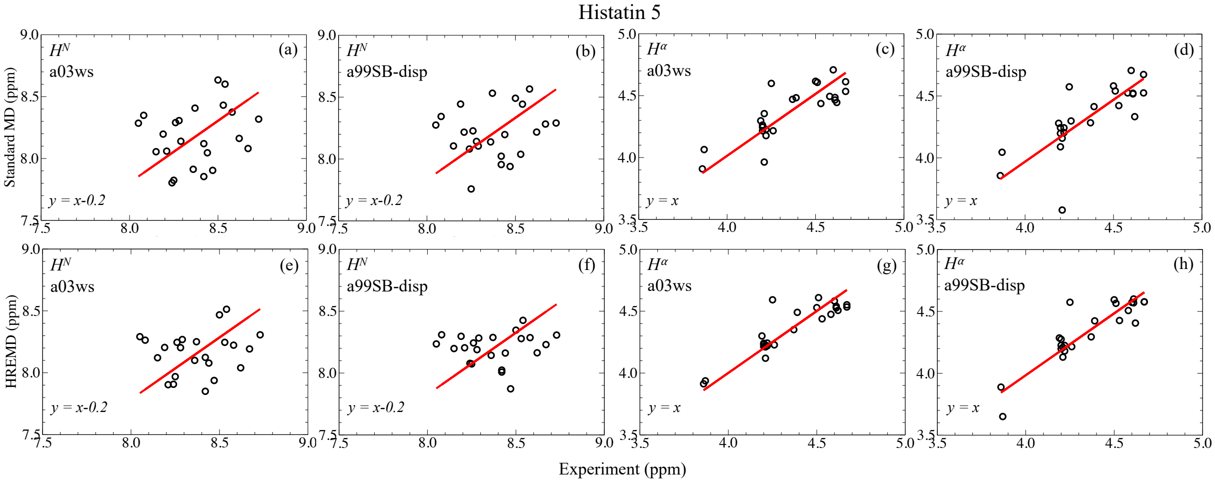

Supplementary Fig. 5. Comparison between the ensemble-averaged calculated and experimental NMR chemical shifts of backbone atoms (H^N and H^α) of Histatin 5.

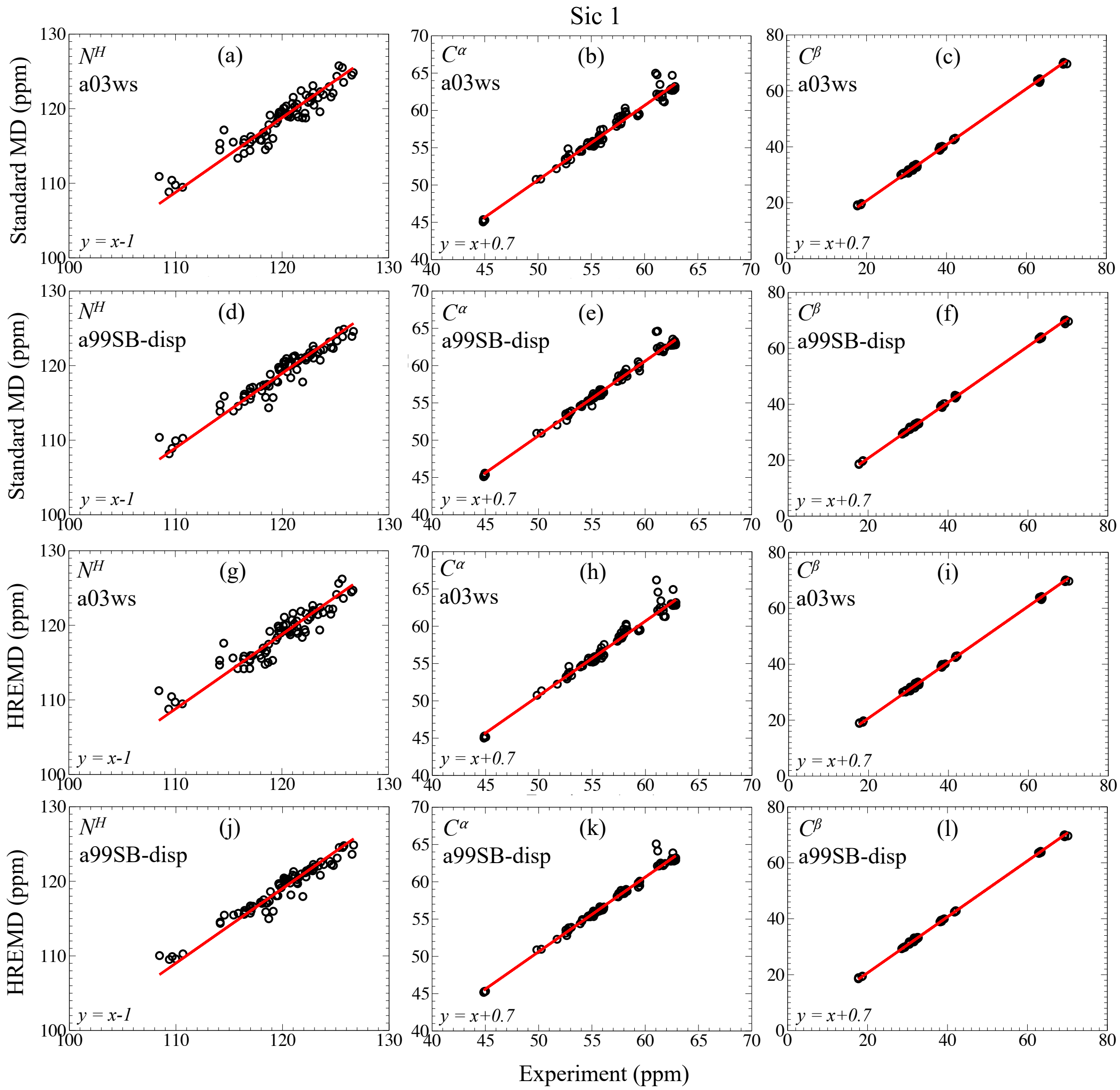

Supplementary Fig. 6. Comparison between the ensemble-averaged calculated and experimental NMR chemical shifts of backbone atoms (N^H, C^α, C^β) of Sic 1.

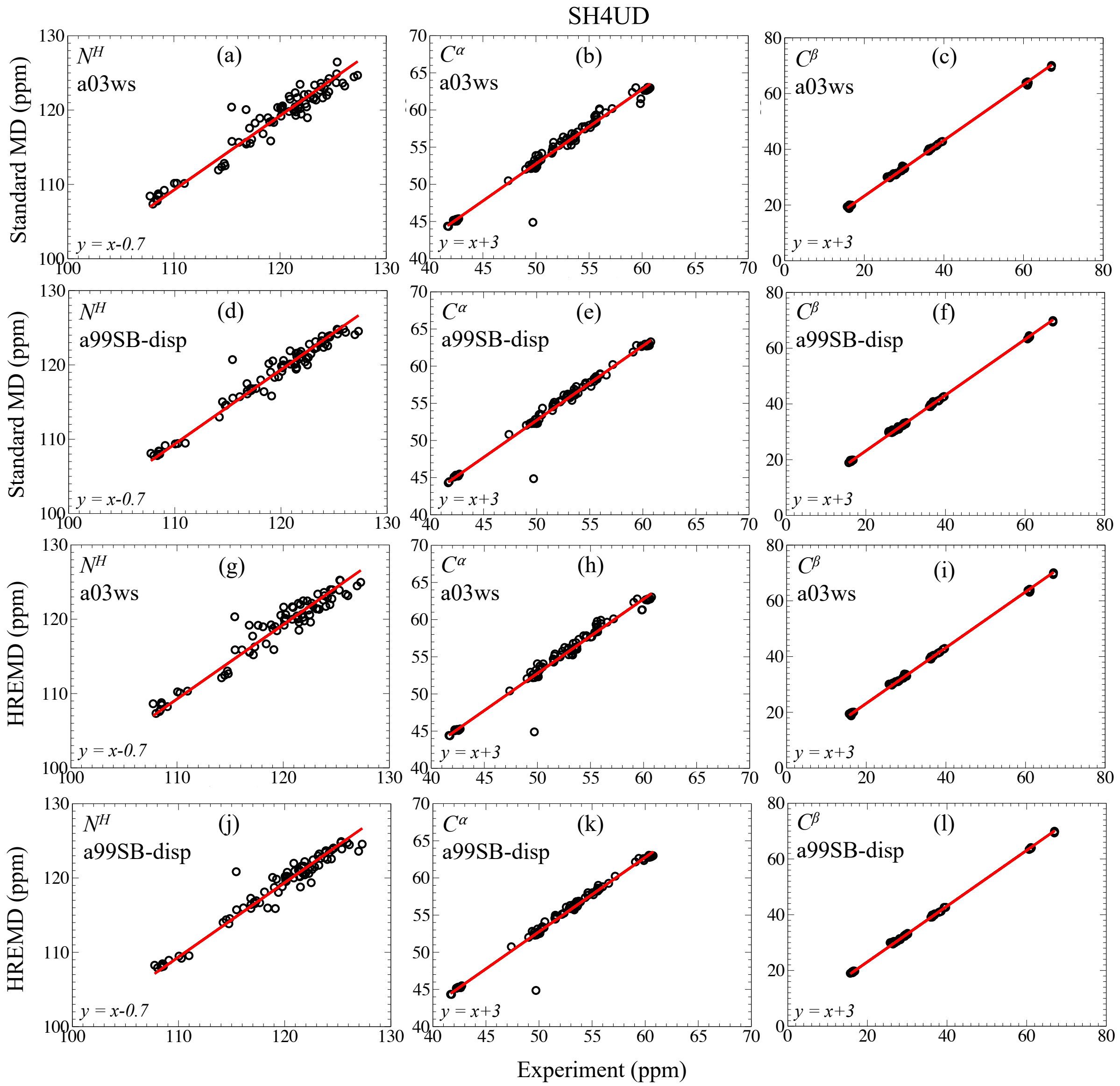

Supplementary Fig. 7. Comparison between the ensemble-averaged calculated and experimental NMR chemical shifts of backbone atoms (N^H, C^α, C^β) of SH4UD.

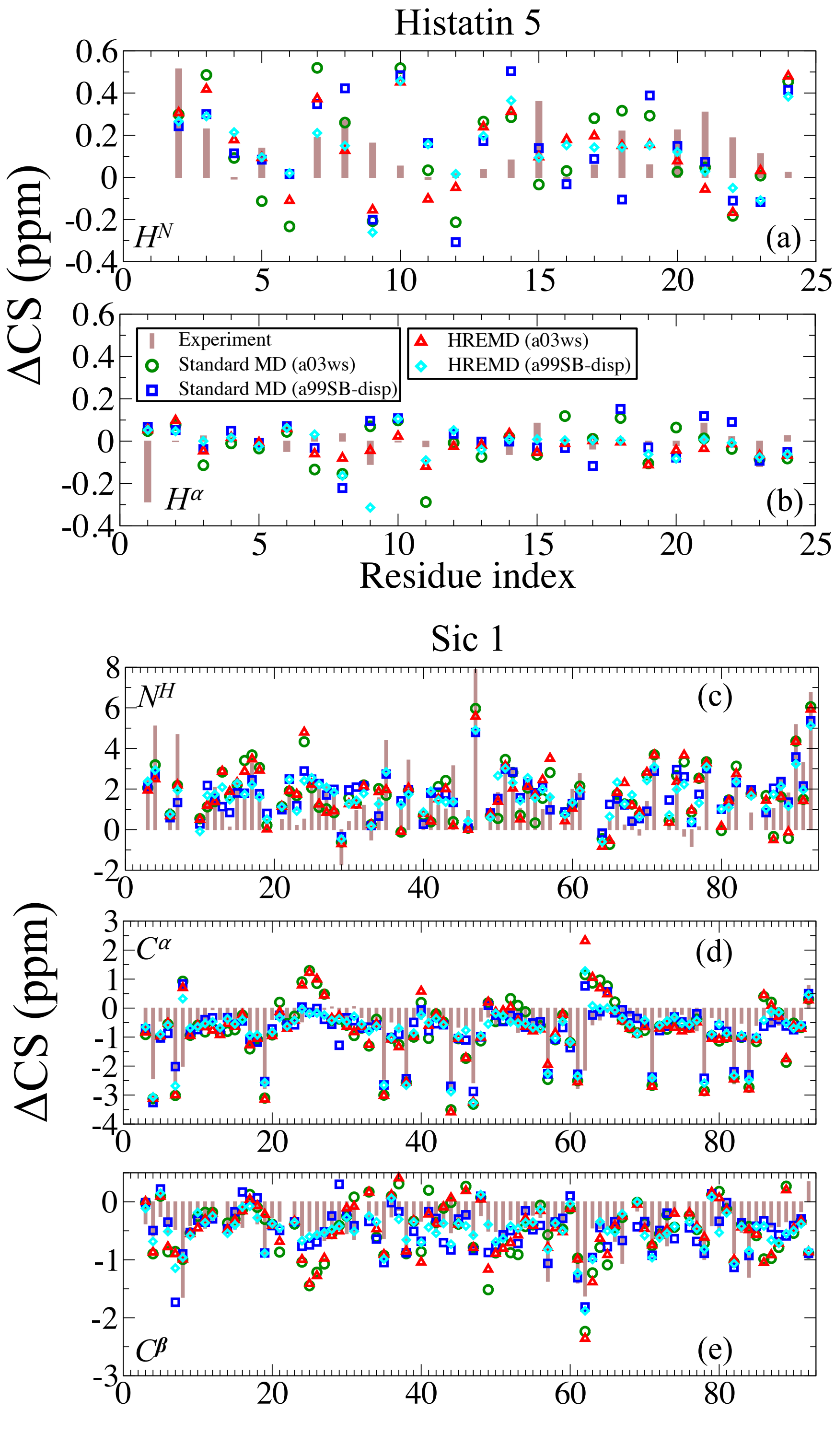

Supplementary Fig. 8. Comparison between the ensemble-averaged calculated and experimental NMR chemical shifts of the Histatin 5 backbone atoms (a) H^N and (b) H^α and Sic 1 backbone atoms (c) N^H, (d) C^α and (e) C^β; The experimental[7,8] and calculated values from MD simulations are shown by different color lines.

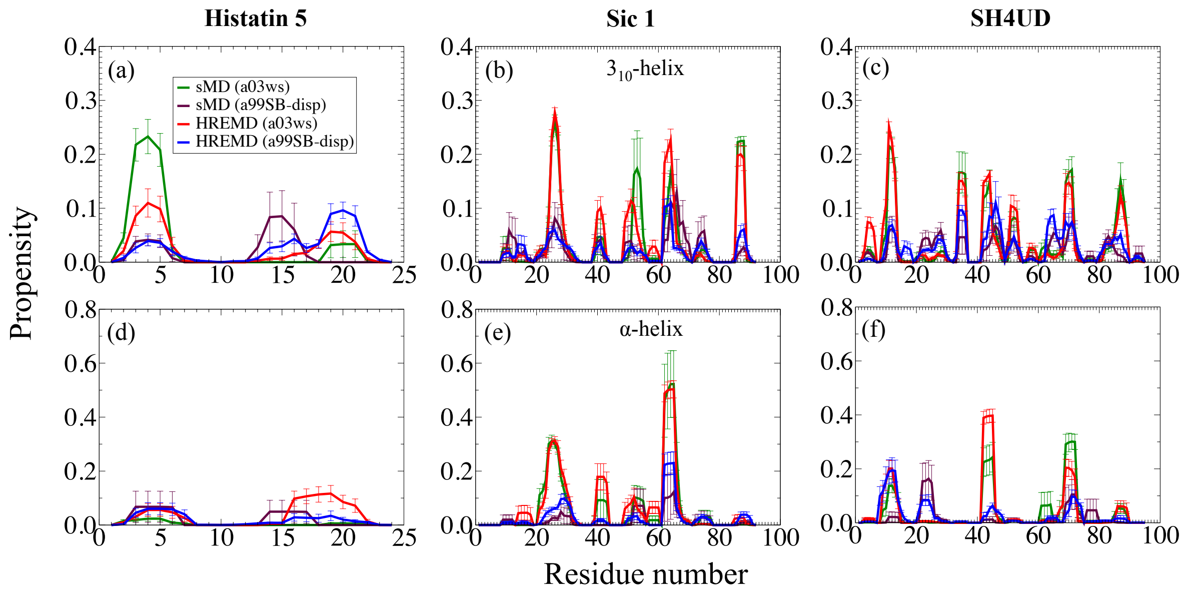

Supplementary Fig. 9. Helical structure propensity. Propensity is scaled between 0 and 1 implying none (0%) and all the snapshot (100%) recorded in MD trajectory respectively here and in the subsequent figures below.

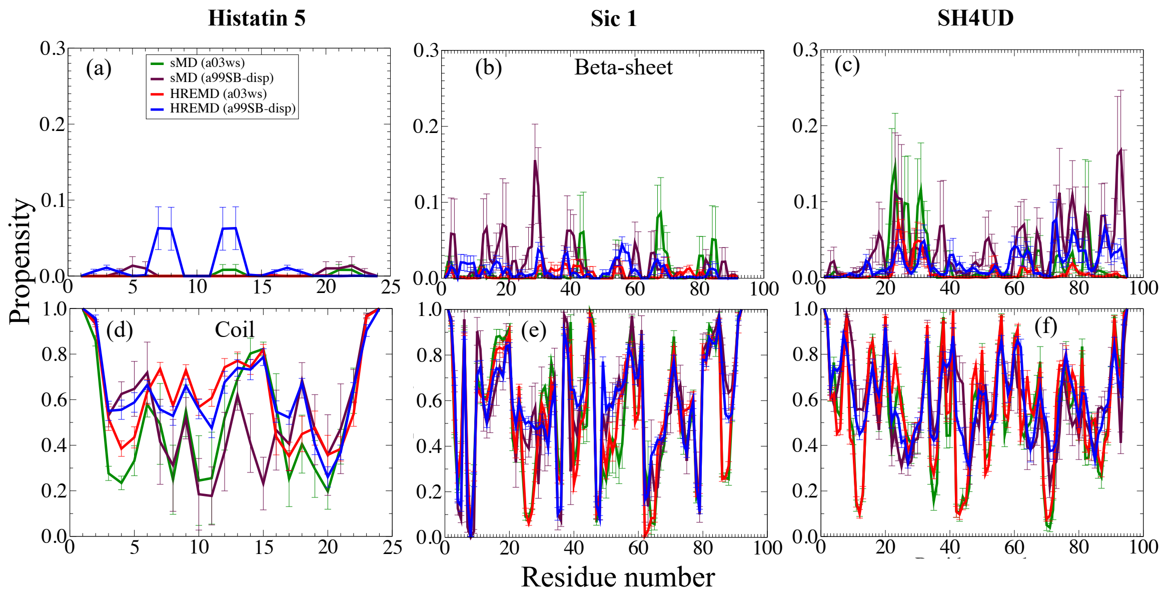

Supplementary Fig. 10. Propensity of β-sheet and coil structures.

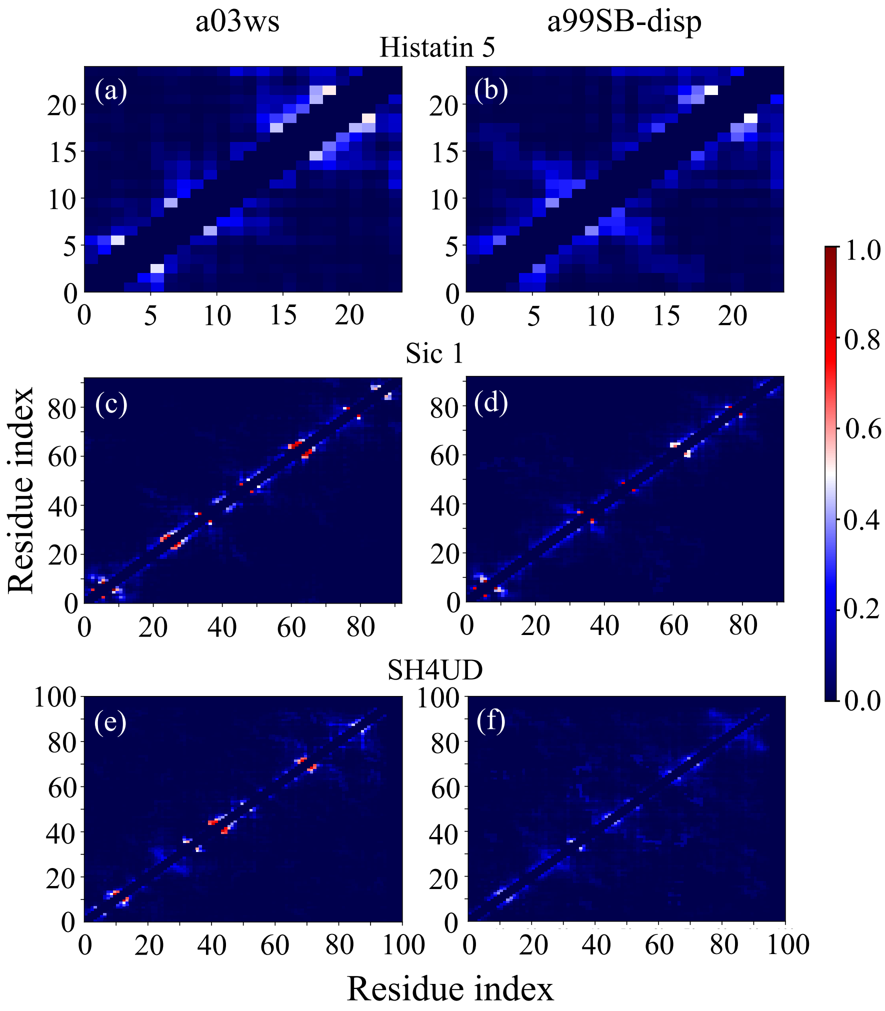

Supplementary Fig. 11. Contact maps of IDPs calculated using Contact Map Explorer (https://contact-map.readthedocs.io/en/latest/index.html). The cut-off distance of 0.45 nm was used and the atoms of 2 residues on either side of given residue and to itself are excluded. The color index represents the fraction of native contacts in HREMD trajectory for each simulation.

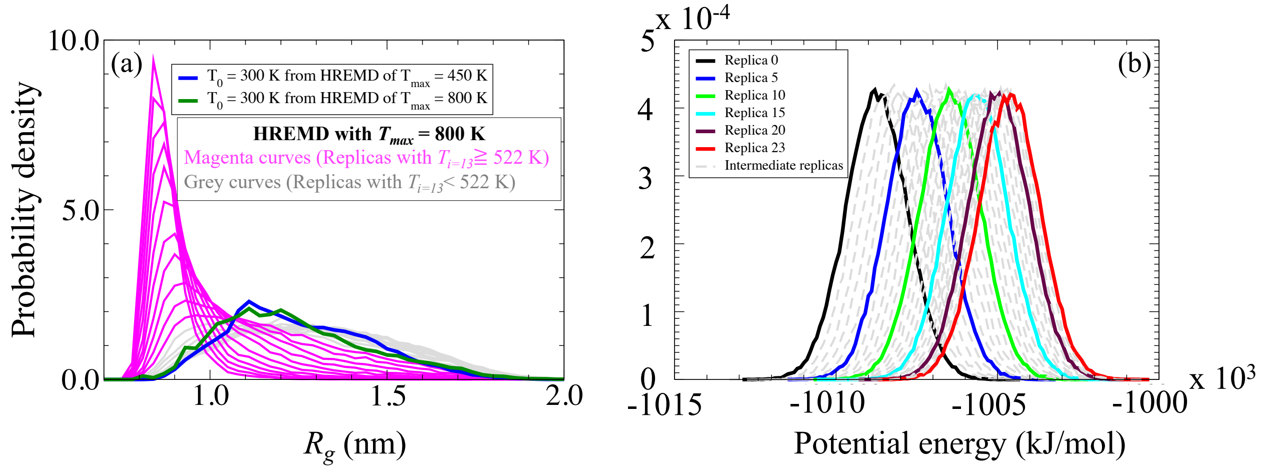

Supplementary Fig. 12. Results from all the replicas of HREMD simulations of Histatin 5 (a99SB-disp) using T_{max} = 800 K. (a) Histograms of R_g of with temperatures above $T_{i=13}$ = 522 K (magenta) generate overly collapsed conformations compared below $T_{i=13}$ (grey). The lowest rank replica (T_0 = 300 K) from HREMD using T_{max} = 800 K is shown in green. For comparison, we show the lowest ranked replica from the T_{max} = 450 K HREMD reported in the main text with blue curve. (b) Potential energy of a system for all the replicas from HREMD with T_{max} = 800 K. A overlap between neighboring replicas confirms that there is no phase transition.

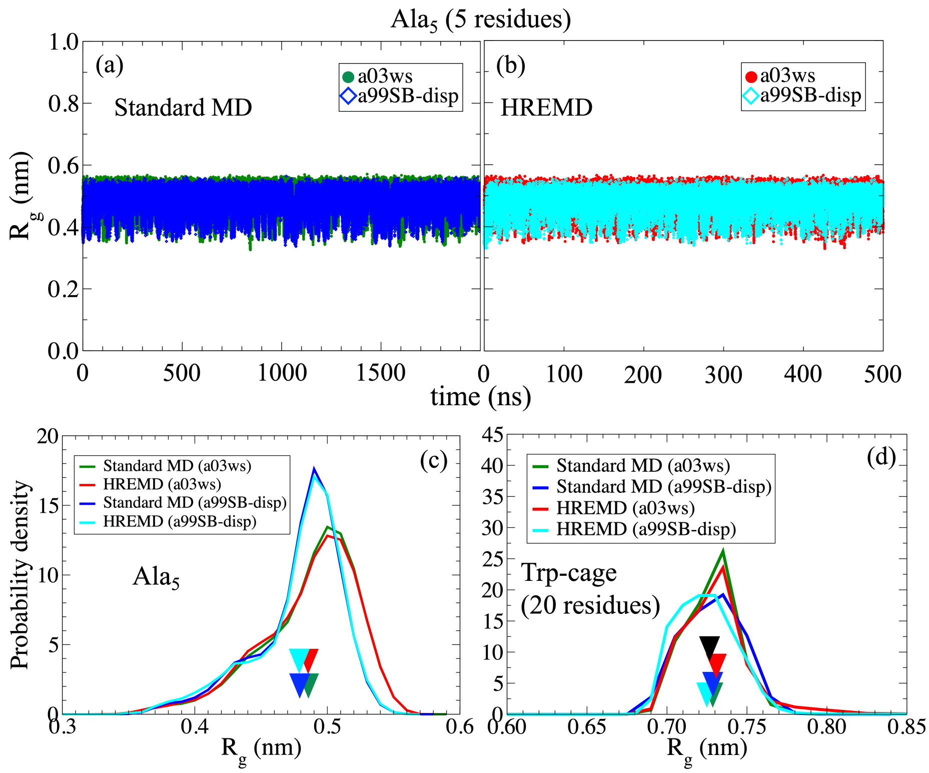

Supplementary Fig. 13. The radius of gyration, R_g vs. time are plotted for (a) standard MD and (b) HREMD trajectories Ala_5. Histograms of R_g (c) Ala_5 and (d) Trp-cage. The HREMD simulations were performed with 4 and 8 replicas for Ala5 and Trp-cage respectively (each replica is 500 ns long). The equivalent lengths of standard MDs were also simulated for comparison (2 μs and 4 μs for Ala_5 and Trp-cage respectively). The inverted triangles indicate the average value of R_g with each color corresponding to force field and sampling method. The black inverted triangle in panel (d) is the average value of R_g of Trp-cage calculated from NMR ensemble (PDB 1L2Y)[9]. The starting structure of Trp-cage for simulations is taken from PDB 1L2Y[9], whereas the initial structure of Ala_5 was constructed using Visual Molecular Dynamics[10].

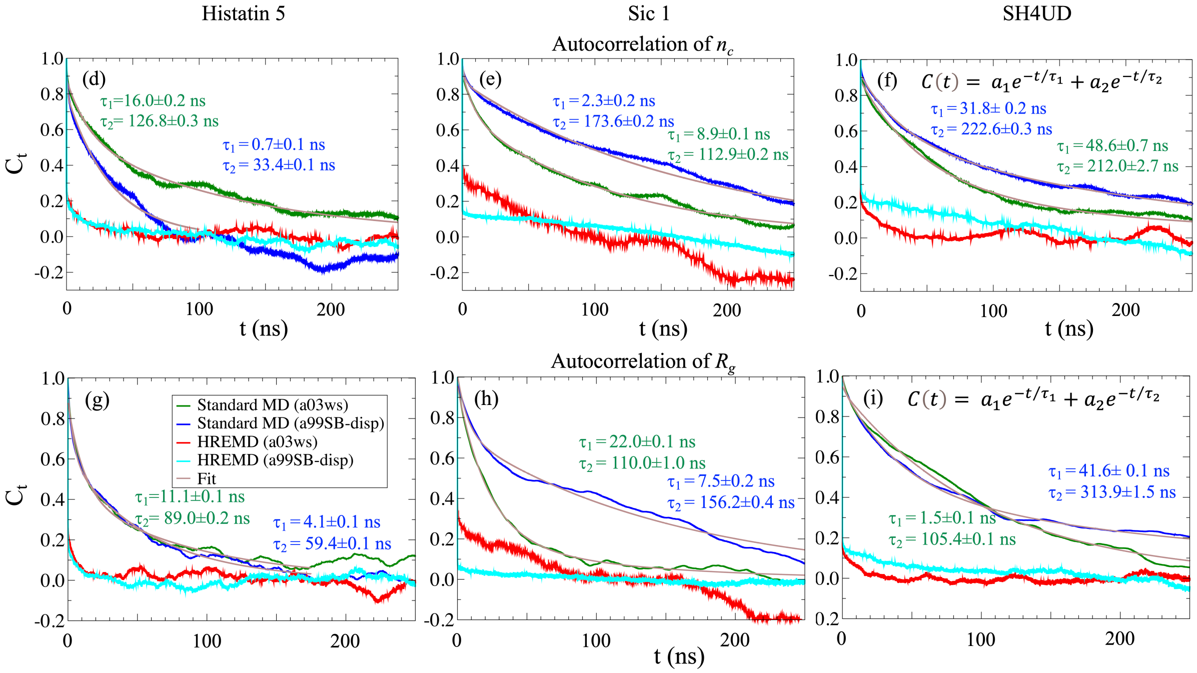

Supplementary Fig. 14. The autocorrelation function of the number of inter-residue contacts (a-c) and of the R_g (d-f). Here, a contact is defined if any two heavy atoms between two residues *i* and *j* are at a distance less than 0.45 nm with | *i* - *j* |>3. The autocorrelation function for the standard MD is fitted with the sum of two exponential functions given $C(t) = a_1e^{-t/\tau_1} + a_2e^{-t/\tau_2}$, where τ_1 and τ_2 are the correlation time constants for faster and slower decay processes, respectively. However, the steep autocorrelations decay from the HREMD could not be fitted with exponential functions.

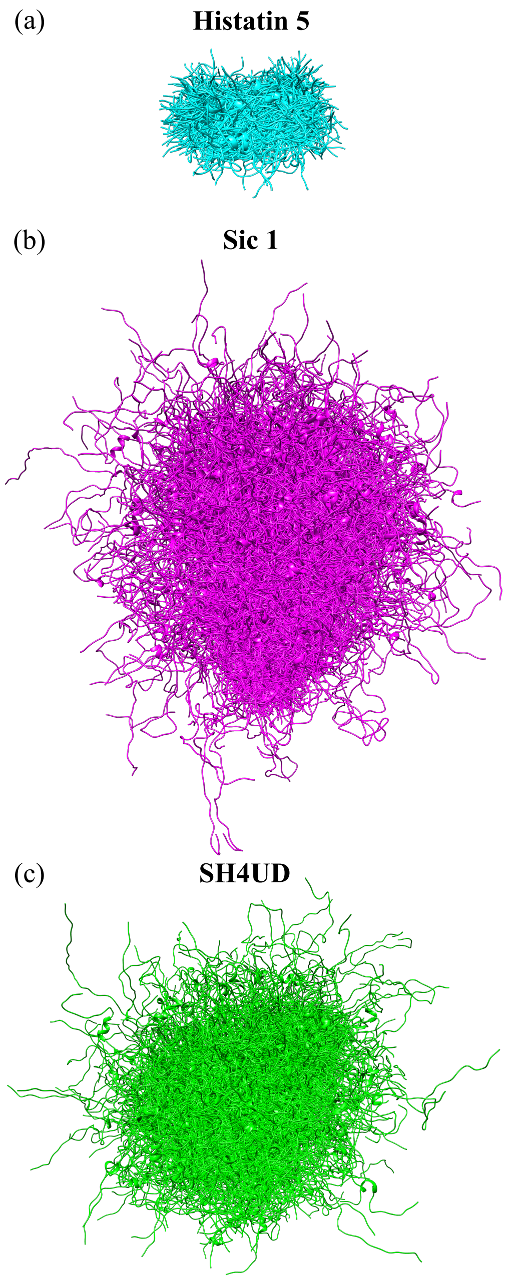

Supplementary Fig. 15. Ensemble of structures of proteins from HREMD simulations. Ensemble obtained from structures saved every 500 ps (total frames 1k) of lowest rank replica of 500 ns long HREMD trajectory (a99SB-disp) of (a) Histatin 5, (b) Sic 1, and (c) SH4UD. The size of the ensemble is not drawn to scale and thus cannot be compared to each other.

Supplementary Table 1. Details of standard MD simulations. Multiple copies were run with different initial velocity distributions.

Proteins	*T* (K)	Force field	Number of atoms	Number of copy x Length of simulation (ns)
Ala_5	298	a03ws	~9k	1x2000
		a99SB-disp		
Trp-cage	282	a03ws	~11k	4x1000
		a99SB-disp		
Histatin 5	300	a03ws	~76k	5x1000
		a99SB-disp		
Sic 1	293	a03ws	~690k	1x3000+3x1700
		a99SB-disp		
SH4UD	300	a03ws	~320k	1x4000+5x1200
		a99SB-disp		1x4000+4x1600

Supplementary Table 2. Details of HREMD simulations. T_0 is the effective temperature of the lowest-rank replica used in the analysis. T_{max} is the effective temperature of the highest-rank replica. p_{ex} is the average exchange probability of the lowest rank replica. Remarks indicate the agreement between the lowest rank replica ensemble with experimental data. The number of atoms in each simulation is the same as that in **Table S1**. The HREMD runs in bold-italics-large font are used to obtain the data shown in the main text.

Proteins	Force field	T_0 (K)	T_{max} (K)	Avg. p_{ex} of T_0 replica	# of replicas (each 500 ns long)
Ala_5	a03ws	298	400	0.4	4
	a99SB-disp		400	0.4	4
Trp-cage	a03ws	282	400	0.4	8
	a99SB-disp		400	0.4	8
Histatin 5	a03ws	300	***425***	***0.4***	***10***
	a99SB-disp		***450***	***0.3***	***10***
			800	0.3	24
Sic 1	a03ws	293	***400***	***0.5***	***16***
	a99SB-disp		***400***	***0.4***	***16***
SH4UD	a03ws	300	***400***	***0.6***	***20***
	a99SB-disp		***450***	***0.5***	***20***

Supplementary Table 3. Scaling factor λ_i- and temperature T_i- of the ith replica for HREMD simulations of IDPs.

**Ala5**		**Trp-cage**	
**T_0=298 K - T_{max}=400 K**		**T_0=282 K - T_{max}=400 K**	
λ_i	T_i (K)	λ_i	T_i (K)
1.00	298.00	1.00	282.00
0.91	328.72	0.95	296.44
0.72	362.61	0.90	311.62
0.74	400.00	0.86	327.58
		0.82	344.35
		0.78	361.98
		0.74	380.52
		0.70	400.00

**Histatin 5**					
**T_0=300 K - T_{max}=425 K**		**T_0=300 K - T_{max}=450 K**		**T_0=300 K - T_{max}=800 K**	
λ_i	T_i (K)	λ_i	T_i (K)	λ_i	T_i (K)
1.00	300.00	1.00	300.00	1.00	300.00
0.96	311.84	0.96	313.82	0.96	313.07
0.93	324.14	0.91	328.29	0.92	326.71
0.89	336.93	0.87	343.41	0.88	340.94
0.86	350.23	0.84	359.24	0.84	355.80
0.82	364.05	0.80	375.79	0.81	371.30
0.79	378.41	0.76	393.11	0.77	387.47
0.76	393.35	0.73	411.23	0.74	404.36
0.73	408.87	0.70	430.18	0.71	421.97
0.71	425.00	0.67	450.00	0.68	440.36
				0.65	459.54
				0.63	479.56
				0.60	500.46
				**0.57**	**522.26 ($T_{collapse}$)**
				0.55	545.01
				0.53	568.76
				0.51	593.54
				0.48	619.40
				0.46	646.38
				0.44	674.54
				0.43	703.93
				0.41	734.60
				0.39	766.60
				0.38	800.00

Sic 1		SH4UD			
T_0=293 K - T_{max}=400 K		T_0=300 K - T_{max}=400 K		T_0=300 K - T_{max}=450 K	
λ_i	T_i (K)	λ_i	T_i (K)	λ_i	T_i (K)
1.00	293.00	1.00	300.00	1.00	300.00
0.98	299.14	0.98	304.58	0.98	306.47
0.96	305.42	0.97	309.22	0.96	313.08
0.94	311.82	0.96	313.94	0.94	319.83
0.92	318.36	0.94	318.73	0.92	326.73
0.90	325.04	0.93	323.59	0.90	333.78
0.88	331.85	0.91	328.53	0.88	340.98
0.86	338.81	0.90	333.54	0.86	348.33
0.85	345.92	0.89	338.63	0.84	355.85
0.83	353.17	0.87	343.80	0.83	363.52
0.81	360.58	0.86	349.04	0.81	371.36
0.80	368.14	0.85	354.37	0.79	379.38
0.78	375.86	0.83	359.77	0.77	387.56
0.76	383.74	0.82	365.26	0.76	395.92
0.75	391.78	0.81	370.84	0.74	404.46
0.73	400.00	0.80	376.49	0.73	413.18
		0.78	382.24	0.71	422.09
		0.77	388.07	0.70	431.20
		0.76	393.99	0.68	440.50
		0.75	400.00	0.67	450.00

Supplementary Table 4. The ensemble-averaged values of R_g of proteins from standard MD and HREMD simulations. R_g is calculated from atomic coordinates using GROMACS in-built tool.

**Proteins**	**FF**	T_0 **(K)**	$R_{g,MD}$ **(nm)**	**HREMD** T_{max} **(K)**	$R_{g,HREMD}$ **(nm)**
Ala_5	a03ws	298	0.487±0.001	400	0.486±0.001
	a99SB-disp		0.479±0.001	400	0.479±0.002
Trp-cage	a03ws	282	0.728±0.005	400	0.732±0.004
	a99SB-disp		0.727±0.005	400	0.725±0.002
	a03ws	300	1.10±0.03	425	1.23±0.02
Histatin 5	a99SB-disp		1.15±0.03	450	1.26±0.01
				800	1.24±0.01
Sic 1	a03ws	293	3.04±0.04	400	3.14±0.13
	a99SB-disp		2.60±0.09	400	3.05±0.04
SH4UD	a03ws	300	2.05±0.14	400	2.60±0.05
	a99SB-disp		2.00±0.07	450	2.50±0.10

Supplementary Table 5. χ^2, defined in **Eq. (5)**, between small-angle scattering experiments and MD simulations averaged over the entire simulation trajectory.

		χ^2		
Sampling method	Force field	Histatin 5 (SAXS)	Sic 1 (SAXS)	SH4UD (SAXS, SANS)
Standard MD	a03ws	5.3	0.2	6.2, 2.0
	a99SB-disp	3.0	3.4	6.8, 2.0
HREMD	a03ws	2.0	0.2	1.2, 1.3
	a99SB-disp	2.0	0.2	1.0, 1.3

Supplementary Table 6. Correlation time estimated from autocorrelation of n_c and R_g such that $C_t = e^{-1} \approx 0.368$ at $t=\tau$.

**IDPs**	**Force fields**	**Correlation time, τ from autocorrelation of n_c (ns)**		**Correlation time, τ from autocorrelation of R_g (ns)**	
		Standard MD	HREMD	Standard MD	HREMD
Histatin 5	a03ws	51	0.01	29	0.01
	a99SB-disp	28	0.02	31	0.01
Sic 1	a03ws	69	0.76	28	0.10
	a99SB-disp	168	0.01	118	0.01
SH4UD	a03ws	66	0.01	101	0.01
	a99SB-disp	106	0.02	99	0.01

Supplementary references.

1 Appel, R. D., Bairoch, A. & Hochstrasser, D. F. A new generation of information retrieval tools for biologists: the example of the ExPASy WWW server. *Trends Biochem. Sci* **19**, 258-260, doi:https://doi.org/10.1016/0968-0004(94)90153-8 (1994).

2 Kyte, J. & Doolittle, R. F. A simple method for displaying the hydropathic character of a protein. *J. Mol. Biol.* **157**, 105-132, doi:https://doi.org/10.1016/0022-2836(82)90515-0 (1982).

3 Best, R. B., Zheng, W. & Mittal, J. Balanced Protein-Water Interactions Improve Properties of Disordered Proteins and Non-Specific Protein Association. *J Chem Theory Comput* **10**, 5113-5124, doi:10.1021/ct500569b (2014).

4 Robustelli, P., Piana, S. & Shaw, D. E. Developing a molecular dynamics force field for both folded and disordered protein states. *Proc Natl Acad Sci U S A* **115**, E4758-E4766, doi:10.1073/pnas.1800690115 (2018).

5 Lindner, B. & Smith, J. C. Sassena - X-ray and neutron scattering calculated from molecular dynamics trajectories using massively parallel computers. *Comput. Phys. Commun.* **183**, 1491-1501, doi:10.1016/j.cpc.2012.02.010 (2012).

6 Marsh, J. A., Singh, V. K., Jia, Z. & Forman-Kay, J. D. Sensitivity of secondary structure propensities to sequence differences between α- and γ-synuclein: Implications for fibrillation. *Protein Sci.* **15**, 2795-2804, doi:10.1110/ps.062465306 (2006).

7 Brewer, D., Hunter, H. & Lajoie, G. NMR studies of the antimicrobial salivary peptides histatin 3 and histatin 5 in aqueous and nonaqueous solutions. *Biochem. Cell Biol.* **76**, 247-256, doi:10.1139/o98-066 (1998).

8 Mittag, T. *et al.* Structure/function implications in a dynamic complex of the intrinsically disordered Sic1 with the Cdc4 subunit of an SCF ubiquitin ligase. *Structure* **18**, 494-506, doi:10.1016/j.str.2010.01.020 (2010).

9 Neidigh, J. W., Fesinmeyer, R. M. & Andersen, N. H. Designing a 20-residue protein. *Nat. Struct. Biol.* **9**, 425-430, doi:10.1038/nsb798 (2002).
10 Humphrey, W., Dalke, A. & Schulten, K. VMD: Visual molecular dynamics. *J. Mol. Graphics* **14**, 33-38, doi:https://doi.org/10.1016/0263-7855(96)00018-5 (1996).
