# Supplementary material for: Full structural ensembles of intrinsically disordered proteins from unbiased molecular dynamics simulations
Source: Commun Biol. 2021 Feb 23;4:243. doi: 10.1038/s42003-021-01759-1 (PMC7902620; doi:10.1038/s42003-021-01759-1)
Supplement: Supplementary file 2 — Description of Supplementary Files [file 42003_2021_1759_MOESM2_ESM.pdf]

Description of Additional Supplementary Files

File name: Supplementary Data 1
Description: The source data of Figure 1. The excel file includes the data of histogram of the radius of gyration, and the theoretical and experimental SAXS intensities for each IDP.

File name: Supplementary Data 2
Description: The source data of Figure 2. The excel file includes the data of NMR secondary chemical shifts (experiment and calculated) and RMSE values for each IDP.

File name: Supplementary Data 3
Description: The source data of Figure 3. The excel file includes the data of orientational correlation function and the average intraprotein pairwise distance for each IDP.

File name: Supplementary Data 4
Description: The "zip" file includes the scripts (codes) and necessary files to run HREMD simulations of IDPs studied in this work.
